# Supplementary material for: A UMLS-based spell checker for natural language processing in vaccine safety
Source: BMC Med Inform Decis Mak. 2007 Feb 12;7:3. doi: 10.1186/1472-6947-7-3 (PMC1805499; doi:10.1186/1472-6947-7-3)
Supplement: Additional file 13 — RAP application directory. Sets up directory for RAP (RDF application for PHP) source code [file 1472-6947-7-3-S13.gz › rap/doc/phpdoc/default/_RdfAPI_php.html]

xml version="1.0" encoding="iso-8859-1"?


Docs for page RdfAPI.php


## /RdfAPI.php

Description

Description |
Includes

- filesource: Source Code for this file

Includes

Description |
Includes

include\_once
(RDFAPI\_INCLUDE\_DIR.PACKAGE\_MODEL)
(line 28)

require\_once
(RDFAPI\_INCLUDE\_DIR.'constants.php')
(line 26)

require\_once
(RDFAPI\_INCLUDE\_DIR.'util/Object.php')
(line 27)

Documentation generated on Fri, 17 Dec 2004 16:16:52 +0100 by phpDocumentor 1.3.0RC3
